# A Low-Cost Implementation of a Potato (*Solanum tuberosum* L.) Moisture Sensor Based on the Howland Current Source Through Discrete Fourier Transform

**DOI:** 10.3390/s25144413

**Published:** 2025-07-15

**Authors:** Laura Giselle Martinez-Ramirez, Juan M. Sierra-Hernandez, Perla Rosa Fitch-Vargas, Julián Andrés Gómez-Salazar, Carolina Bojórquez-Sánchez, Arturo Alfonso Fernandez-Jaramillo

**Affiliations:** 1 Departamento de Ingeniería Electrónica, División de Ingenierías Campus Irapuato-Salamanca, Universidad de Guanajuato, Carretera Salamanca-Valle de Santiago km 3.5 + 1.8 km, Comunidad de Palo Blanco, Salamanca C.P. 36885, Guanajuato, Mexico; lg.martinezramirez@ugto.mx (L.G.M.-R.); jm.sierrahernandez@ugto.mx (J.M.S.-H.); 2 Facultad de Ciencias del Mar, Universidad Autónoma de Sinaloa, Paseo Clausen S/N, Col. Los Pinos, Mazatlán C.P. 82000, Sinaloa, Mexico; perlafitch@uas.edu.mx; 3 Departamento de Alimentos, División de Ciencias de la Vida, Campus Irapuato-Salamanca, Universidad de Guanajuato, Ex-Hacienda El Copal, Carretera Irapuato-Silao km 9, Irapuato C.P. 36500, Guanajuato, Mexico; julian.gomez@ugto.mx; 4 Unidad Académica de Ingeniería en Tecnología Ambiental, Universidad Politécnica de Sinaloa, Carretera Municipal Libre km. 3 Mazatlán-Higueras Colonia Genaro Estrada, Mazatlán C.P. 82199, Sinaloa, Mexico; cbojorquez@upsin.edu.mx; 5 Unidad Académica de Ingeniería Biomédica, Universidad Politécnica de Sinaloa, Carretera Municipal Libre km 3 Mazatlán-Higueras Colonia Genaro Estrada, Mazatlán C.P. 82199, Sinaloa, Mexico

**Keywords:** electrical impedance spectroscopy (EIS), *Solanum tuberosum* L., Howland source, FFT

## Abstract

The growing demand for the production of food has led to the development of new analytical techniques in the food industry, enabling innovative strategies to streamline food production and ensure its physicochemical and microbiological quality. In this work, a smart sensor was developed using the electrical impedance spectroscopy (EIS) technique. The system is based on discrete Fourier transform (DFT) and incorporates a Howland current source. The experimental results showed that the sensor was able to detect the moisture content in potatoes (*Solanum tuberosum* L.). Favorable responses were obtained by exciting the system with two frequency intervals: 0–100 Hz and 500–5000 Hz. An exhaustive analysis of the frequency response was performed to identify the most linear behavior in the moisture measurement, with an R-squared of 0.786 and signals in intervals from 500 to 5000 Hz. Moreover, the linearity remained stable across most frequencies, resulting in consistent measurements, even with the implementation of low-cost components.

## 1. Introduction

Currently, quality standards in the food industry are higher than ever, and regulations concerning exportation and marketing are becoming increasingly stringent. Additionally, the rise in food production necessitates the assurance of its physicochemical and microbiological quality. However, conventional chemical analyses are time-consuming, costly, and often destructive. Therefore, the food industry must develop new techniques to verify food quality [1].

According to the FAO for 2017, the potato (*Solanum Tuberosum* L.) is the third most important food crop globally for human consumption, with a production of 378 million tons and an estimated cultivation area of 19 million hectares worldwide [2]. Therefore, understanding its chemical composition is essential. The main component of this tuber is water, comprising approximately 75% of its weight [3]. Thus, accurately estimating the moisture content is crucial to ensure food quality, consistency, and freshness; to comply with export regulations; and to improve production and processing techniques, such as drying [4]. Additionally, in the food industry, moisture content is one of the main quality indicators. For example, in the production of fried foods, the final average moisture content is inversely proportional to the final fat content, which affects both the flavor and structure of the product. Moreover, moisture content influences the growth of microorganisms in food: a high moisture content promotes their growth, while a low moisture level suspends or delays the metabolic activity of the microorganisms responsible for food spoilage [5].

As mentioned above, conventional methods used to determine the chemical composition of food are typically time-consuming, destructive, and costly. One such method for determining moisture content in food is the gravimetric method. In this approach, the moisture content of a sample is calculated based on its weight loss after being dried in an oven under specific time and temperature conditions. The oven may operate using forced convection, vacuum, or microwave heating [1]. Moreover, this method requires homogeneous samples of a specific size and is destructive. Additionally, during hot-air drying, vegetables undergo changes in their nutrient content, flavor, mechanical properties, and other physical and chemical characteristics. However, it has been reported that, in order to standardize indirect methods, such as electrical impedance spectroscopy (EIS), it is necessary to use direct methods due to their precision [6].

In recent years, different types of smart sensors have been proposed to detect biological and chemical parameters; as a result, they have found broad applications in the food industry. Techniques to determine moisture in food, such as EIS, allow for the analysis of a material’s electrical properties by inducing alternating electrical signals at different frequencies and measuring the response signals [7]. As a non-invasive, rapid, and cost-effective technique, EIS shows significant potential for applications in the food industry [8]. Through real-time impedance spectroscopy, it is possible to evaluate the effects of temperature on potato and starch cell walls and thus, on texture properties [9]. This technique is also useful in the analysis of plant heat stress [10]. Moreover, it has been applied to specific measurements of salt levels in foods such as pork meat [11], and it serves as an effective method for detecting adulterants in food [12]. In addition, the application of EIS to determine moisture content has been reported to ensure optimal quality during the storage of onions and corn [13,14]. Likewise, EIS is employed to identify the most sensitive parameters and to use moisture content as a variable for distinguishing between viable and non-viable seeds in snap bean (*Phaseolus vulgaris* L.) [15].

On the other hand, implementing an EIS analysis system requires a reliable current source. There are different topologies available; however, the type that offers the most suitable characteristics for bioimpedance applications is the Howland current source, as the improved Howland circuit provides high efficiency, a simple structure, high accuracy, and stable injected current [16].

Building on the above, the contribution of new techniques for food analysis can be recognized, with electrical impedance being well-suited for this type of application. However, in most cases, implementing this technology requires precise, large, and costly laboratory equipment. Therefore, this equipment is not suitable for the rough environment of a food processing plant [17]. Consequently, this work proposes the low-cost implementation of a smart sensor based on the Howland circuit. To achieve this, it is first necessary to implement the digital signal synthesis to excite the system and enable spectral analysis. Excitation is applied in a conventional and controlled manner using a sinusoidal signal whose frequency varies over time. This approach enables spectral enrichment, producing signals with greater complexity and richer information for analysis. For this purpose, the system must include a digital-to-analog converter (DAC) to generate an excitation signal at the sample input. Then, through an analog-to-digital converter (ADC), the response signal is acquired and stored in a CSV file, where a database of biochemical response can be created for subsequent analysis. Since the main goal of spectral analysis is to decompose the signal into frequency components, the implementation of digital signal processing techniques, such as Fourier transform, is effective for these purposes, as it enables the observation of the signal’s frequency distribution. The spectral components of the response signal can be corelated with the moisture content of the sample, allowing for characterization according to the moisture percentage in the food.

### 1.1. Bioimpedance

To implement bioimpedance, it is necessary to model the biological system as an electrical equivalent. These electrical properties vary depending on the cellular or compound structures. In the case of a cell, the membrane is composed of a thin lipid bilayer with permeable ion channels, which gives it both capacitive and resistive characteristics [18].

Electrical impedance (Z) is defined as the opposition to alternating electrical currents through a material. It can be represented as a function of two components or vectors: resistance (R) and reactance ($X_{c}$). Therefore, the mathematical representation is complex and can be expressed as Equation (1), where Z is the impedance, R is the resistance, and $X_{c}$ is the reactance.

(1) $$Z=R+jX_{C}$$

Resistance represents the real part and reactance the imaginary part of the impedance. Unlike reactance, resistance does not vary with frequency due to its passive nature. On the other hand, impedance can also be represented in a parallel configuration, as shown in Equation (2), where Y is the admittance, G is its conductance, C is its capacitance, and ω is its angular frequency.

(2) Y=G+jωC

**Figure 1:**
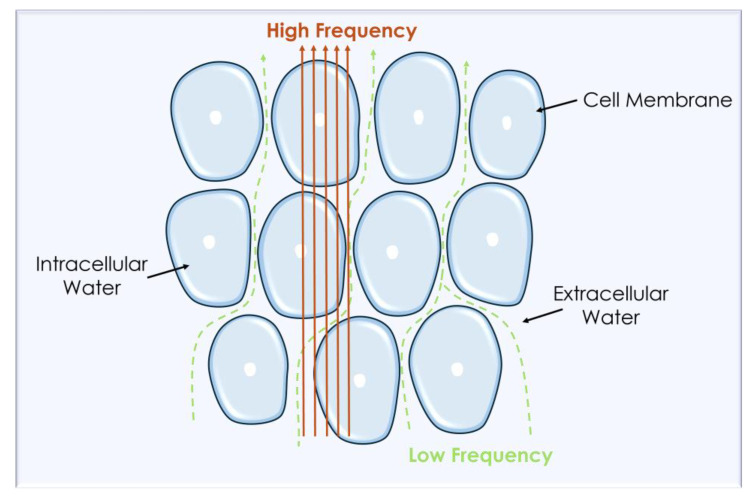
Distribution of high and low frequencies in cell suspension.

Resistance is determined by the flow of current through intracellular and extracellular electrolyte solutions, while reactance depends on the dielectric properties of tissues or the temporary accumulation of charges on cell membranes [19]. Thus, electric current flows through both extracellular and intracellular fluids in different ways; the current with high frequency flows through these two, while low-frequency current cannot pass through the cell membrane [20], as shown in Figure 1.

According to Ohm’s law, V=Z∗I, where Z is the impedance, V is the voltage, and I is the current. Therefore, a controlled current is injected into the sample, and the resulting voltage provides a signal for subsequent analysis [21]. As mentioned above, a database of biochemical response signals was obtained, and digital signal processing (DSP) techniques were implemented. DSP refers to the mathematical representation, algorithms, and techniques used to transform or enhance a signal for a specific purpose [22].

### 1.2. Discrete Fourier Transform

The Fourier series and the Fourier transform are useful for these purposes, as they allow for the visualization of a signal’s frequency distribution. This technique makes it possible to analyze the frequency content using a controlled source signal, such as a chirp waveform, to excite the sample. The resulting response signal is then acquired and used to determine the frequency at which the sensor measurement is most stable [23].

An algorithm called Fast Fourier Transform (FFT) streamlines the implementation of the discrete Fourier transform in digital signal processing, which is a much faster alternative to discrete transform; it is based on the simultaneous solution of linear equations by the method of correlation, an alternative that often improves the efficiency of processing times on the order of hundreds of times.

In this technique, it is necessary to understand that the domain of time and frequency is contained in a signal of N complex numbers in a complex notation. Each of the points mentioned above is made up of a real part and an imaginary part. The FFT operates by decomposing a time domain signal of N points into N time domain signals, each composed of a single point. The second step is calculating N points of the frequency spectrum corresponding to those N points of the signal in the time domain. Finally, the spectrum is synthesized into a single frequency spectrum [23].

This digital signal processing technique is widely implemented in impedance measurement systems, such as humidity sensing, to test the detection characteristics of polymer moisture sensors, where the amplitude and phase of the impedance measurement is based on discrete Fourier transformation (DFT) [24]. There is an analysis of a measurement probe for a high impedance spectroscopy analyzer; the developed analyzer uses a phase-sensitive detector based on the DFT for the determination of the orthogonal parts (real and imaginary) of the measurement signals [25]. The analysis uses Equations (3) and (4) for calculating the DFT. Where x[i] is the time domain signal being analyzed, and $ReX\left[k\right],$ $ImX\left[k\right]$ are the frequency domain signals being calculated. The index  i runs from 0 to N−1, while the index k runs from 0 to N/2 [22].

(3) $$Re\;X\left[k\right]=\sum_{i=0}^{N-1}x\left[i\right]\;cos\;(2\pi ki/N)$$

(4) $$Im\;X\left[k\right]=-\sum_{i=0}^{N-1}x\left[i\right]\;sin\;(2\pi ki/N)$$

## 2. Materials and Methods

The experimental sample consisted of potatoes (*Solanum tuberosum* L.) obtained from a local market and stored in plastic bags. In the laboratory, the samples were washed with tap water to remove adsorbed soil particles, rinsed with distilled water, and then peeled prior to drying.

To implement the moisture smart sensor based on the Howland source, it was essential to include a DC power supply for the Howland source circuit. The electrodes (Delmhorst 30-E/C, ABQ Industrial, The Woodlands, TX, USA) used with this circuit consisted of two insulated pins, which performed well on food samples. Additionally, the measurement system employed a multifunctional instrument (Digilent Analog Discovery 2, Digilent, Austin, TX, USA), which generated the excitation signal in our system and obtained the output signal to display on a computer for subsequent analysis using digital signal processing techniques.

### 2.1. Gravimetric Analisys

First, to characterize the sensor, gravimetric measurements were performed on the food samples. This technique is used to determine the mass or concentration of total water in a sample by measuring weight loss under specific time and temperature conditions. Therefore, this method was used as a reference for determining the moisture content in potatoes (*Solanum tuberosum* L.). The materials used for the gravimetric analysis included a convection oven, an analytical balance, desiccators, crucible tongs, and aluminum trays.

**Figure 2:**
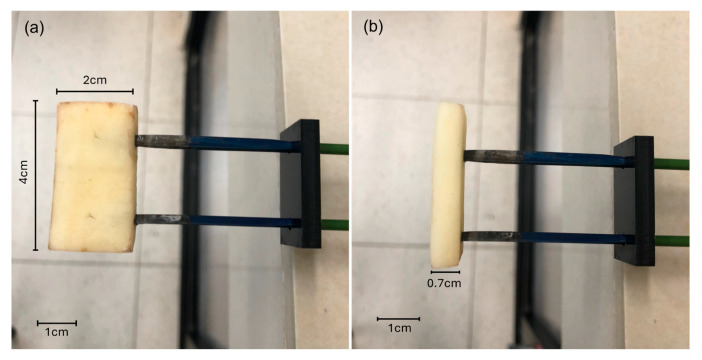
Sample size (**a**) front view and (**b**) side view of the sample placed on the electrodes.

The gravimetric method was implemented following the AOAC standard [26]. The trays were dried in the oven at 105 °C for 24 h to remove moisture and obtain a constant weight. Afterward, the aluminum trays were placed in desiccators to bring them to room temperature and were weighed using an analytical balance. The potato samples were cut into rectangles measuring 2 cm × 4 cm × 0.7 cm, (Figure 2), deposited in the aluminum trays, and placed in the drying oven at 50 ± 1 °C for 24 h. Finally, the samples were removed from the oven, placed in desiccators for 20 min, weighed again, and the weight loss was used to calculate the partial moisture content. The percentage of moisture was calculated using the gravimetric analysis Formula (5):

(5) $$\%\;moisture=\left(\frac{\left(Aw+S\right)-(Aw+Ds)}{\left(S\right)}\right)*100$$

where Aw is the aluminum tray weight, S is the weight of the sample, and Ds is the dry sample.

### 2.2. Howland Current Source

**Figure 3:**
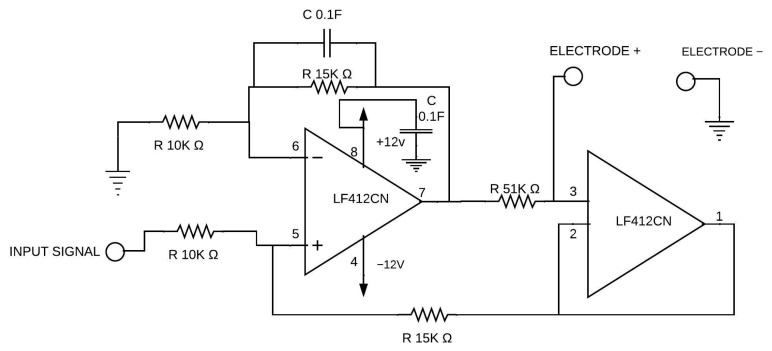
Diagram of the Howland current source circuit.

In order to perform an electrical bioimpedance analysis, it was necessary to implement a voltage-controlled current source (VCCS), specifically the Howland source. The circuit configuration is shown in Figure 3. This circuit uses an operational amplifier with both inverting and non-inverting feedback (LF412CN Instrumentation amplifier, Texas Instruments, Dallas, TX, USA), along with 10 k Ω and 51 k Ω precision resistors and a 0.1 F ceramic capacitor to prevent unwanted oscillations in the circuit. It is also important to note that the current source must display a relatively high impedance to allow variations in load impedance without affecting the load current.

### 2.3. Characterization of the Sensor

As mentioned above, the moisture content in the potato samples was determined through gravimetric analysis. This value was used as a reference for the subsequent sensor measurements.

**Figure 4:**
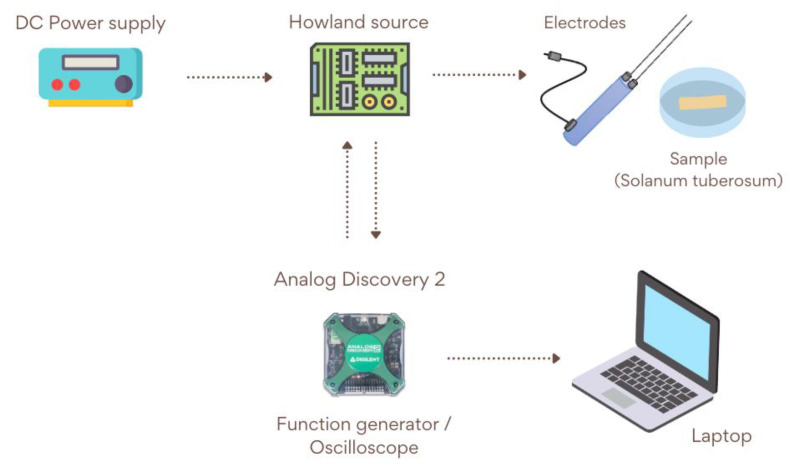
The moisture sensor schematic diagram.

Initially, the Howland source circuit was powered with a ±12V DC supply. The electrodes were connected to the potato sample. Subsequently, using the Analog Discovery device and in order to perform more detailed frequency analysis, two different set of frequency signals were used to excite the sample. Additionally, due to the multifunctionality of this device, it was also used as an oscilloscope. Using it, the output signal was acquired and later processed through digital signal processing techniques to detect changes in the spectral content. This allowed the samples to be characterized according to their partial moisture content. For the system block diagram, see Figure 4.

**Figure 5:**
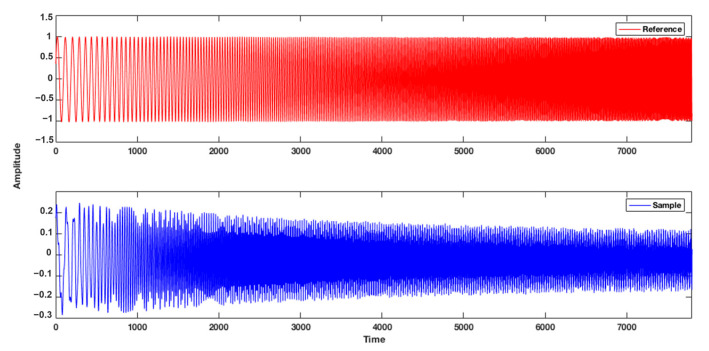
Reference signals and the moisture output of the sample.

**Figure 6:**
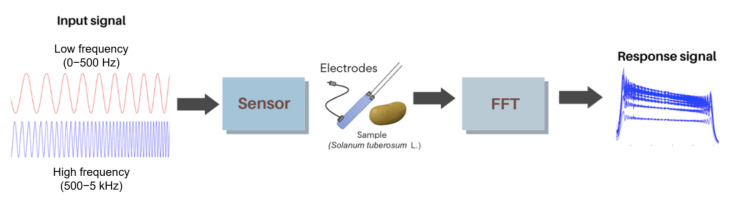
Methodology diagram of the moisture sensor.

Subsequently, measurements were conducted using the sensor based on the Howland source, in which chirp signals were applied in two frequency ranges: 0 to 500 Hz and 500 to 5 kHz, respectively. As shown in Figure 5, the reference signal and the response signal were obtained from the sensor when one of the potato (*Solanum tuberosum* L.) samples was tested. In this response, a change in amplitude, associated with their corresponding frequencies, is observed at different time instants. Therefore, digital signal processing techniques will be applied to enable a more meaningful analysis of the output signal. The block diagram of the measurement system is shown in Figure 6.

## 3. Results

### Experimental Results

In order to verify the validity of the sensor based on the Howland source, several experiments were conducted. These experiments were designed to measure the moisture in potato (*Solanum tuberosum* L.), and the spectral analysis was performed using the FFT. As mentioned above, for the characterization of the sensor, it was necessary to obtain a sufficient number of measurements. To achieve this, a large batch of potatoes was used, totaling 130 samples of *Solanum tuberosum* L. The samples were measured on different days and at time intervals of 2, 4, and 6 h in a convection oven to produce a variety of partial moisture percentages.

**Figure 7:**
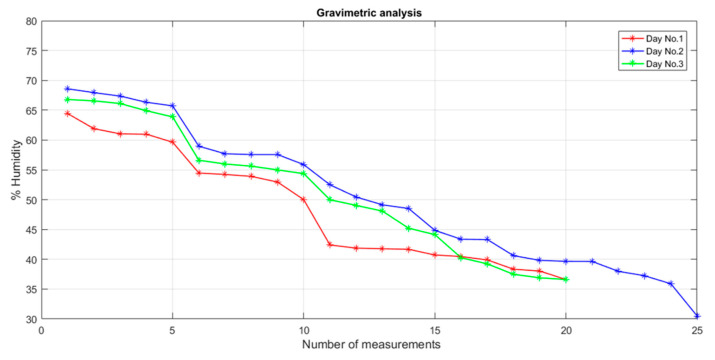
Gravimetric analysis results.

Moisture measurements were carried out in triplicate over three days of testing. It is important to note that the initial tests were not excluded from the analysis; instead, they were used to calibrate the sensor by adjusting the measurement procedure. On day one, the moisture percentage of 20 potato samples was obtained using the gravimetric method. In the first five samples, total moisture was determined to serve as a reference for subsequent measurements, while partial moisture was measured in the remaining 15 samples. The partial moisture percentages ranged from a minimum of 36.60% to a maximum of 64.50%. On day two, 25 samples were analyzed to broaden the range of the moisture values. A minimum partial moisture of 30% and a maximum of 68.5% were recorded, levels which were similar to the results from the first day. Finally, on day three, measurements were obtained from 20 samples. As with the previous tests, the results fell within similar moisture ranges, which is favorable for comparing the sensor’s output. Figure 7 shows the results, ordered from the highest to the lowest partial moisture percentage.

## 4. Discussion

**Figure 8:**
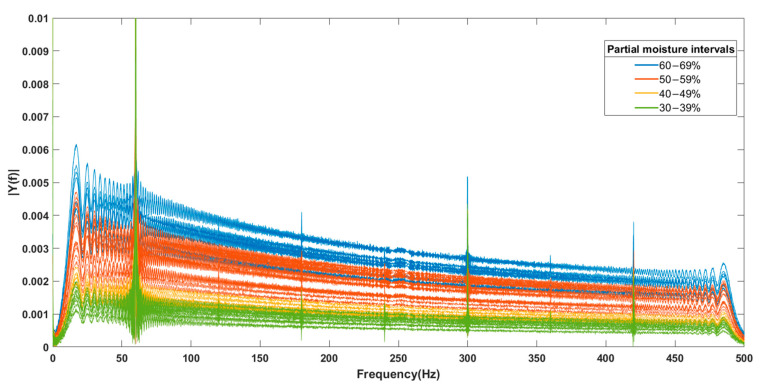
FFT of measurements obtained for low frequency signals.

As mentioned above, a low-frequency chirp signal was first used in the range of 0 to 500 Hz in 65 samples subjected to different drying intervals over the three days of testing. The digital signal processing technique applied was the FFT, which was used to analyze the output signals from the sensor. These signals were sorted according to their partial moisture percentages and grouped into categories of 30, 40, 50, and 60% partial moisture. As shown in Figure 8, a favorable trend was observed in relation to the partial moisture percentage; the output spectra exhibited a characteristic amplitude response. Similar to the previous analysis, measurements were performed using the sensor with a high-frequency chirp signal, specifically in the range of 500 to 5 kHz.

In addition, it is important to note that we are proposing a new and low-cost implementation method for measuring moisture. However, since this method lacks a reference, it is necessary to assess its repeatability. To provide confidence in the results, a sigma rule analysis was performed to evaluate the repeatability of the moisture percentage measurements over the three days of testing. This analysis was based on the data presented in Figure 7.

**Figure 9:**
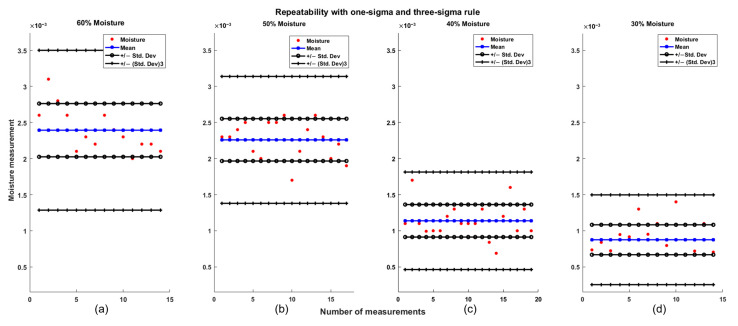
Repeatability test using one-sigma and three-sigma rule for measurements with a low-frequency response for (**a**) 60% moisture, (**b**) 50% moisture, (**c**) 40% moisture, and (**d**) 30% moisture.

First, the analysis focused on samples showing the response at low frequencies, specifically in the most stable region of the signal: between 185 and 235 Hz in the FFT output. The mean and standard deviation were calculated based on all measurements from days 1, 2, and 3, totaling 65 observations. As shown in Table 1 and Figure 9, the repeatability results indicate that all four groups comply with the three-sigma rule, as 100% of the measurements fall within acceptable limits. Furthermore, the one-sigma rule is also satisfied, since more than 70% of the measurements are within this range, which exceeds the 68% required threshold [27,28].

**Figure 10:**
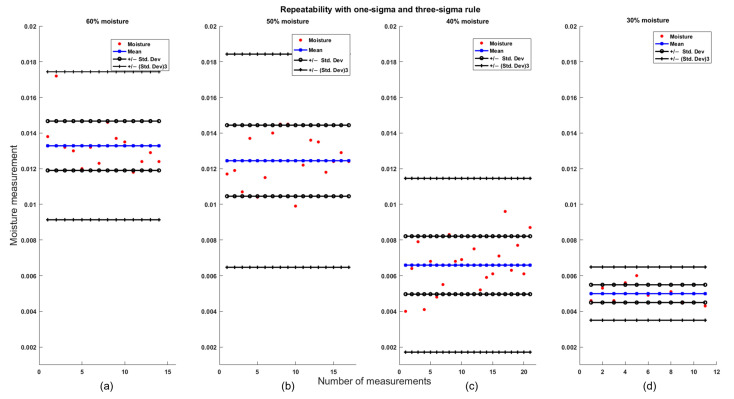
Repeatability test using one-sigma and three-sigma rule for measurements with a high-frequency for (**a**) 60% moisture, (**b**) 50% moisture, (**c**) 40% moisture, and (**d**) 30% moisture.

In addition, the repeatability test was carried out for the samples with responses at high frequencies. This analysis was based on 63 measurements obtained from tests conducted on days 1, 2, and 3, within the 1.5 to 3 kHz interval of the FFT output signal. As shown in Table 2 and Figure 10, favorable results were also achieved. According to the one-sigma rule, more than 70% of the measurements fell within acceptable limits, and under the three-sigma rule, 100% of the measurements were within the expected range.

In addition to the above, in order to design a smart sensor system, it is important to ensure linearity in the measurements. Therefore, a linearity analysis was performed at each individual frequency, correlating the partial moisture percentage with the corresponding FFT amplitude.

**Figure 11:**
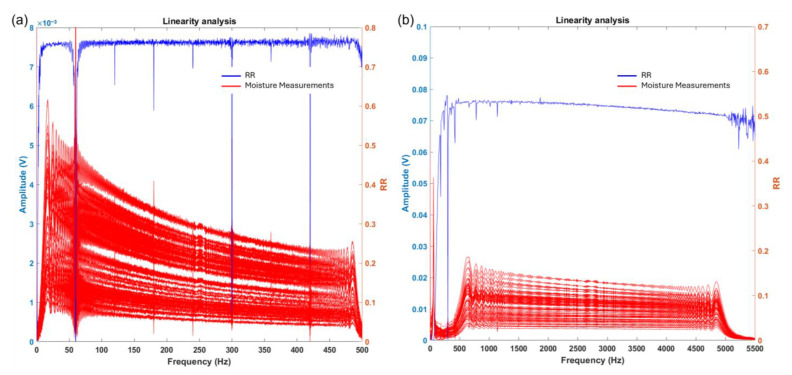
Linear analysis of (**a**) low-frequency and (**b**) high-frequency signals.

At each frequency, the linearity analysis was used to identify which frequencies are most suitable for further evaluation, as shown in Figure 11. The result of the linearity analysis is represented by the blue line. The R-squared value is a statistical measure of how closely the data fit the regression line.

**Figure 12:**
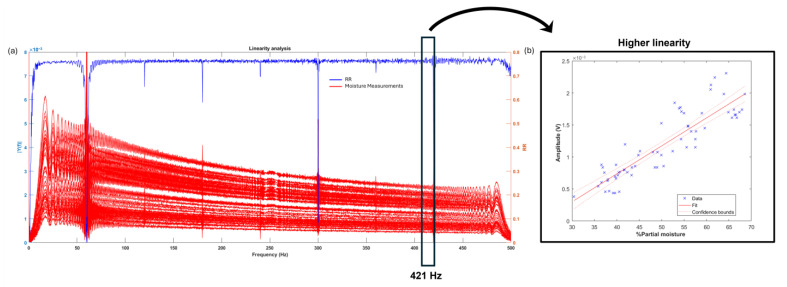
(**a**) Linear frequency of low-frequency signals; (**b**) higher linearity.

For the low-frequency signals, the R-squared value is 0.786, and for the high-frequency signals, it is 0.529. The most linear frequency is identified in Figure 12, with a value of 421.02 Hz for the low-frequency analysis and 1868.8 Hz for the high-frequency analysis. As shown in the figure, the highest linearity is observed in the low-frequency analysis, as more experimental data points fall within the confidence bounds.

**Figure 13:**
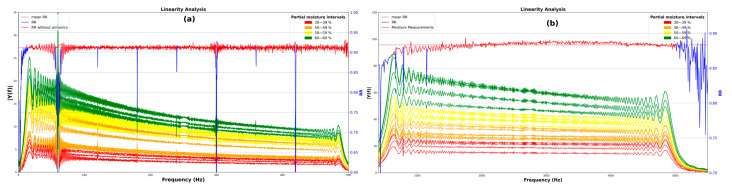
Linearity analysis of a single side of the sample: (**a**) lower frequencies, (**b**) higher frequencies.

It is important to note that all previous analyses were performed using measurements taken from all sides of the sample. This approach aimed to provide a more robust statistical analysis. However, in practical applications, only one side of the sample is typically measured. Therefore, Figure 13 presents the linearity analysis for both low and high frequencies based solely on measurements from a single side of the sample. Unlike the previous measurements, when analyzing only one side, the linearity becomes more consistent and improves, yielding an average R-squared result of 0.9114 for low frequencies and 0.8834 for high frequencies, as indicated by the red dashed line. It is also important to note that harmonics that are multiples of the 60 Hz of electrical noise were excluded, as shown by the difference between the blue and red R-squared lines.

## 5. Conclusions

This work describes a robust methodology for developing a low-cost, moisture-smart sensor implementation. It proposes new techniques for real-time food analysis, with electrical impedance spectroscopy identified as a suitable tool for this type of application. This is possible because the R-squared values remain relatively constant across the frequency range, allowing for the use of an excitation source implemented with low-cost components, since a highly stable excitation frequency is not required. Despite this, the measurement remains reliable.

Therefore, this low-cost smart sensor is presented for measuring moisture in potato (*Solanum tuberosum* L.), operating by injecting a current into the sample and measuring its voltage response, making a current source essential, and the Howland source provides adequate characteristics for such applications.

In addition, working with low-frequency signals in the 0 to 500 Hz range proved to be the most suitable for moisture determination, showing a linearity of R-squared = 0.786 and a very low standard deviation, which supports the R-squared values and enables the design of a moisture sensor within this range.

This type of sensor improves food moisture measurement by providing faster results compared to the time required for conventional methods and without wasting samples. Consequently, the use of such techniques in food analysis enables innovative strategies with broad applications in the food industry, offering opportunities for the development and implementation of smart sensors for rapid, real-time food quality analysis.

## Figures and Tables

**Table 1:** Repeatability results for low-frequency responses.

% Partial Moisture	Mean	Std. Dev.	% Inside One-Sigma
60	0.0024	3.6918 × 10^−4^	78.58
50	0.0023	2.9307 × 10^−4^	76.47
40	0.0011	2.2492 × 10^−4^	78.94
30	8.7561 × 10^−4^	2.0715 × 10^−4^	71.42

**Table 2:** Repeatability results for high-frequency responses.

% Partial Moisture	Mean	Std. Dev.	% Inside One-Sigma
60	0.0133	0.0014	85.72
50	0.0124	0.0020	76.47
40	0.0066	0.0016	71.43
30	0.0050	0.0004968	72.72

## Data Availability

The data presented in this study are available upon request from the corresponding author.

## Abbreviations

The following abbreviations are used in this manuscript:

EIS	Electrical Impedance Spectroscopy
DFT	Discrete Fourier Transform
DAC	Digital-to-Analog Converter
ADC	Analog-to-Digital Converter
DSP	Digital Signal Processing
FFT	Fast Fourier Transform
